# Physical Activity in Relation to Wellbeing Among Newly Arrived Refugees in Sweden: A Quantitative Study

**DOI:** 10.3389/fpubh.2020.532883

**Published:** 2021-01-08

**Authors:** Katarina Sjögren Forss, Elisabeth Mangrio, Matti Leijon, Mathias Grahn, Slobodan Zdravkovic

**Affiliations:** ^1^Department of Care Science, Faculty of Health and Society, Malmö University, Malmö, Sweden; ^2^Malmö Institute for Studies of Migration, Diversity and Welfare (MIM), Malmö University, Malmö, Sweden; ^3^Department of Medical and Health Sciences, Linköping University, Linköping, Sweden; ^4^Unit for Statistics and Data, Municipality of Malmö, Malmö, Sweden

**Keywords:** health, migration, physical activity, refugees, wellbeing

## Abstract

**Background:** Little is known about physical activity among newly arrived refugees and what impact physical activity might have on their health, as measured by mental wellbeing, vitality, stress and sleep quality. Thus, this study sought to investigate the relationship between physical activity and wellbeing among refugees who were newly arrived in Sweden.

**Methods:** The present study was based on the results from a survey, conducted in 2015–2016 among newly arrived adult refugees who spoke Arabic, Pashto, Somali or Dari, participated in a mandatory public integration support programme in the Scania region of Sweden and agreed to participate in the survey. Ultimately 681 participants completed the survey (a response rate of 39.5%).

**Results:** We found a significant association between physical activity and mental wellbeing, vitality, stress and sleep quality among newly arrived refugees.

**Conclusions:** Newly arrived refugees need to be informed about the importance of prioritizing physical activity for their health and wellbeing, regardless of their external circumstances, and supported in their attempts to do so.

## Introduction

Refugees comprise a vulnerable group in several ways and they are frequently exposed to significant health-related risk factors. Good health is essential not only for everyday life (1) but also for refugees' abilities to integrate themselves into their new societies and labor markets (2). Refugees' health-related situations behaviors must therefore be addressed from multiple perspectives.

Previous studies have shown that many refugees suffer from complex mental health needs (3, 4) such as those caused by trauma, war or violence in their home country and difficult and traumatic journeys to their new host countries, which can cause depression, anxiety and post-traumatic stress disorder (5, 6). Refugees are also frequently exposed to stressful situations after arriving in their host countries such as lengthy asylum processes, poor living conditions, and uncertainty about their future and these can have additional negative impacts on their health and wellbeing (7–9). Previous research has also shown that insomnia, potentially as a result of these circumstances, is common among refugees; moreover, insomnia is also often correlated with a variety of depressive symptoms (10).

Physical activity (PA) has a well-documented positive impact on health (11); it can prevent or delay the onset of a variety of mental disorders and may also have therapeutic benefits when used as alone or as adjunct treatment (12). PA significantly enhances wellbeing (13) and individuals who are regularly physically active has been found to have a higher life satisfaction as compared to those who are inactive (14–16). Evidence also suggests that moderate-to-vigorous PA ameliorate the quality of sleep in adults by reducing the length of time it takes to go to sleep and reducing the time one is awake after going to sleep and before rising in the morning. The time in deep sleep can also increase as a result of PA as well as a reduction of daytime sleepiness (17).

However, studies about PA among refugees are sparse. A few studies have found that the benefits of PA were well known among refugees, but that the biggest barrier to their participation was lack of familiarity and comfort with taking the first steps toward PA in their new host country (18, 19). A majority of refugees in one study also stated that they were less physically active since arriving to the host country than before they fled their home countries (18); it is plausible that newly arrived refugees feel that they have more important priorities than PA (20). In a recent study from Sweden that aimed to explore trauma-afflicted refugees' participation in PA as part of their treatment it was found that the refugees experienced that PA relieved their mental distress (21). As a consequence of being more physically active, they reported an increased wellbeing that for example could be related to that the felt more resilient and less stressed, had more energy and a better sleep. They also experienced PA as a positive interruption to their daily life (21). Moreover, a study among female Somali refugees in New Zealand found that PA also improved refugees' development of social relationships and community cohesion in their new home country (22). Studies by Ollif (23) as well as by Spaaij (24) have confirmed these findings. PA may therefore be an effective way for refugees to improve their mental health by helping them cope with circumstances in their new host countries as well as with their memories from their past.

In the light of recent global increases in refugee populations, it is likely that the incidence of both physical and mental health disorders among refugees will also increase, with consequent challenges for public health. It is therefore crucial to investigate refugees' participation in PA and the impact that PA can have on refugees' health and wellbeing, however to the best of our knowledge, few studies have explored this topic to date. The present study therefore sought to investigate the relationship between PA and wellbeing among newly arrived refugees in Sweden. Wellbeing was defined here as the balance point between an individual's available resources and challenges (physiological, social, physical) (25).

## Materials and Methods

### Participants

All newly arrived (range: a few months to 2 years) adult refugees in the Scania region of Sweden, who spoke Arabic, Pashto, Somali or Dari, who held either a temporary or permanent residence permit, and who participated in the obligatory public integration support program for refugees (MILSA) between February 2015 and February 2016 were asked to participate in the study. As they had applied for asylum and were granted either temporary or permanent residence permits, they had obtained refugee status. The MILSA platform is a research-based support platform for migration and health and include a close collaboration between Malmö University and the county government in Scania. MILSA is a part of Partnership Scania which is governed by the Regional Agreement (RÖK) for enabling an effective integration of migrants (26).

Data collection was conducted through a self-administered questionnaire, distributed by the civic and health communicators, and included questions about health, sleep, education level, wellbeing, housing accommodations, social relationships, work and access to healthcare. The questionnaire was translated from Swedish to Arabic, Pashto, Somali and Dari by authorized translators. Approximately, 1,700 questionnaires were distributed.

### Measures

#### Dependent Variables

We assessed four dependent variables: sleep quality, stress, mental health, and vitality.

- Sleep quality was assessed by the question: *How do you sleep in general?* The answers were grouped into “good” or “poor”.
- Stress was assessed by the question: *Do you feel stressed in your daily life?* The answers were grouped into “yes” or “no”.
- Mental health was derived by the General Health Questionnaire (GHQ) 12 scale (27).
- Vitality was assessed by the vitality scale from The Short Form Health Survey Questionnaire (SF-36) (28).

Low vitality was measured by SF 36 (28) from the following four questions: *Did you feel full of pep? Did you have a lot of energy? Did you feel worn out?* and *Did you feel tired?* During the past 4 weeks. For each question, the respondent was asked to give the one answer that comes closest to the way he/she has been feeling. The response alternatives provided were: *All of the time*; *Most of the time*; *A good bit of the time*; *Some of the time*; *A little of the time* and *None of the time*. The questions *Did you feel full of pep?* and *Did you have a lot of energy?* were recoded so that a low score indicated a negative outcome on all four included questions. Mean value was calculated on all answered questions (at least two of the four questions had to be answered). Sum = 4 × the mean value was produced. The sum was indexed 0–100. A cutoff value corresponding to the respondent on average answering one of the two worst response alternatives gave a low vitality.

#### Independent Variables

We also assessed four independent variables: PA, education levels, age, and gender. Sine current guidelines stipulate that, in order to receive significant health benefits from PA, adults should engage in at least 150 min per week of moderate intensity, or 75 min per week of vigorous intensity aerobic activity (29), we assessed participants' PA using two questions, designed to assess high-intensity and moderate-intensity PA respectively: (1) *During a regular week*, how *much time do you spend engaging in physical exercise that causes you to be out of breath, such as running, exercise or ball sport?* And (2) *During a regular week, how much time do you spend engaging in everyday physical activities, such as walking, cycling or gardening?*

*The* first *question* had six possible answer choices: 0 min/no time; <30 min; 30–60 min (0.5–1 h); 60–90 min (1–1.5 h); 90–120 min (1.5–2 h) 2 h or more; *the second question* had seven possible answer choices: Calculate all time (at least 10 min at a time). Zero minute/No time; <30 min; 30–60 min (0.5–1 h); 60–90 min (1–1.5 h); 90–150 min (1.5–2.5 h); 150–300 min (2.5–5 h); and more than 300 min (5 h). Each participant's responses to the two questions were then weighted and combined into a common measure called “activity minutes” in which the time from high- intensity activities were doubled (e.g., 45 min walking + 45 min running = 135 activity min) (30). The reference group for the analyses was >300 min per week.

Independent variables also included education levels, age and gender. Education levels were categorized as high (>12 years), medium (10–12 years) or low (<9 years). Age was divided into five groups (18–34, 35–44, 45–54, 55–64, or 65–80) and was measured as a continuous variable, but for descriptive statistics. Gender was listed as either female or male.

#### Statistical Analysis

We used descriptive statistics to evaluate participants' sample characteristics, which were calculated as frequencies and percentages. Logistic regression was used to analyse the association between the dependent variables and the independent variable PA by calculating the odds ratios (OR) and 95% confidence intervals (CI). Multiple logistic regression was used to calculate adjusted OR and 95% CI for the influence of education levels, age, and gender as possible confounding variables. *P*-values below 0.05 were considered statistically significant. All statistical analyses were conducted using SPSS Statistics 22 for Windows (IBM Corporation, Armonk, New York, United States) was used for the analysis.

## Results

In total, 681 newly arrived refugees speaking Arabic or Dari answered the questionnaire (response rate 39.5%). Sociodemographic information of the participants is presented in Table 1.

**Table 1:** Participant characteristics.

**Gender**	***N* (%)**
	**Total 681**
Male	461 (68)
Female	204 (30)
Not reported	16 (2)
**Age**	
18–34	307 (45)
35–44	155 (23)
>45	219 (32)
**Education level**	
High education level (>12 years of school)	301 (44)
Medium education level (10–12 years of school)	141 (21)
Low education level (<9 years of school)	146 (21)
Not reported	93 (14)

The crude analyses resulted in significant association between all levels of PA and all wellbeing parameters except for poor mental wellbeing and PA of 150 and 299 min per week (presented in Table 2). The OR of poor sleeping resulted in 2.2 (95% CI 1.2–4.0) for those with a PA of 0 min/week and in 2.5 (1.4–4.4) for those with 1–149 min/week of PA. The OR of stress resulted in 3.9 (95% CI 2.1–7.2) for those with a PA of 0 min/week and in 2.3 (95% CI 1.3–3.9) for those with 1–149 min/week of PA. The OR of poor mental wellbeing resulted in 4.5 (95% CI 2.3–8.6) for those with a PA of 0 min/week and in 2.2 (1.2–4.0) for those with 1–149 min/week of PA. The OR of low vitality resulted in 5.0 (95% CI 2.2–11.5) for those with a PA of 0 min/week and in 2.4 (95% CI 1.3–4.7) for those with 1–149 min/week of PA. When adjusting these findings for age, gender, and level of education all significant OR remained significant (presented in Table 3). The non-significant crude association between poor mental wellbeing and PA of 150 and 299 min/week remained non-significant after adjusting for age, gender and educational level. Adjusting for these parameters increased the majority of the OR confirming the unadjusted results and increasing the risk that low levels of PA has on stress, poor mental wellbeing, and low vitality. For poor mental wellbeing, low vitality and stress the results indicates a trend meaning that the lower level of PA the higher risk for theses outcomes. However, this trend cannot be considered as significant.

**Table 2:** Correlations between physical activity levels and sleep, mental wellbeing, vitality and stress, given as crude odds ratios (ORs).

	**Sleep**		**Mental wellbeing**		**Vitality**		**Stress**	
	**OR**		**OR**		**OR**		**OR**	
Level of physical activity^*^	**95% CI**	***P*****-value**	**95% CI**	***P-*****value**	**95% CI**	***P-*****value**	**95% CI**	***P*****-value**
150–299 min/week	1.706	0.027	1.628	0.066	1.711	0.044	2.149	0.002
	(1.149–3.079)		(0.969–2.378)		(1.015–2.885)		(1.337–3.452)	
1–149 min/week	2.498	0.001	2.217	0.008	2.446	0.007	2.266	0.004
	(1.436–4.345)		(1.228–4.005)		(1.283–4.662)		(1.305–3.936)	
0 min/week	2.235	0.007	4.454	<0.001	5.027	<0.001	3.929	<0.001
	(1.247–4.006)		(2.316–8.566)		(2.197–11.503)		(2.135–7.228)	

* The reference group for all analyses was >300 min per week.

**Table 3:** Correlations between physical activity levels and sleep, mental wellbeing, vitality and stress, given as adjusted odds ratios (ORs) for age, gender and education levels.

	**Sleep**		**Mental wellbeing**		**Vitality**		**Stress**	
	**OR**		**OR**		**OR**		**OR**	
Level of physical activity^*^	**95% CI**	***P*****-value**	**95% CI**	***P*****-value**	**95% CI**	***P*****-value**	**95% CI**	***P*****-value**
150–299 min/week	1.881	0.012	1.558	0.1	1.749	0.039	2.323	0.001
	(1.149–3.079)		(0.919–2.639)		(1.029–2.972)		(1.412–3.820)	
1–149 min/week	2.826	<0.001	2.41	0.005	2.483	0.007	3.043	<0.001
	(1.581–5.051)		(1.310–4.436)		(1.284–4.804)		(1.691–5.475)	
0 min/week	2.504	0.003	4.814	<0.001	5.051	<0.001	5.426	<0.001
	(1.355–4.628)		(2.454–9.444)		(2.176–11.722)		(2.818–10.448)	

* The reference group for all analyses was > 300 min per week.

## Discussion

Our findings suggest that there is a significant association between both sleep quality, mental wellbeing, vitality, and stress in relation to PA among newly arrived refugees. Consequently, encouraging participation in PA among refugees should be a priority from a public health perspective, as we know from previous research that many live in challenging conditions (7–9) that might have a negative impact on their health. Although PA cannot change refugees' living conditions, its ability to positively affect their health and wellbeing should not be underestimated and from a public health standpoint, newly arrived refugees should be encouraged to participate in PA. As we also know that there can be a decline in PA after arriving to the host country as compared to the previous PA level (19), it is important to find health promoting arenas and actors that can promote PA among refugees.

Participants in the present study took part in a public integration support program that is mandatory for all newly arrived refugees in Sweden. The program seeks to provide a basic understanding of Swedish society and a solid foundation for participants continued knowledge acquisition (26). As such, it provides an existing, practical framework within which PA opportunities could be offered to newly arrived refugees (31). For example, the programme could include information about the importance of PA and even integrate PA within the programme itself. Our findings demonstrated the importance of increasing refugees' awareness of the benefits of PA from both health and social and integration perspectives. By using a participatory approach, and thus allowing individuals to voice their perceptions to PA is important to understand and improve their health behavior and have in previous research (18) shown to be valuable in capturing refugees' perceptions of PA behavior.

It is also important to bear in mind that perceptions of PA may vary between cultures. Receptivity to PA is strongly influenced by an individual's beliefs, experiences and group identity, and it is therefore important to identify both the challenges that limit individual participation and the factors that enable it. For example, one review among culturally and linguistically diverse groups of migrants to Western societies found that environmental and socioeconomic factors, cultural or religious beliefs and issues with social relationships could all function as limiting factors of PA (32). Also, refugee's lack of knowledge about PA's benefits, as well as lack of role models who engaged in PA has been found to limit their participation in PA (33); in contrast, refugees who had social support for PA from family, friends, and their community were more physically active and expressed greater motivation for PA (18).

Thus, it is important to design PA respectfully and sustainably according to the needs of diverse groups and to the needs of the individual (34). Countries and communities should strive to create environments and conditions that enable PA, that has a potential as a calming influence, relaxation and a non-threatening social environment which can thus be a vital part to a community building process for refugees but might also promote sleep habits as well as psychological wellbeing and prevent stress. However, more research in the field is needed. Also, in most international models of refugee health, psychosocial interventions are highlighted, however few guidelines exist as to the implementation of specific programmes, for example how to promote PA.

## Strengths and Limitations

The findings of the present study should be considered in the context of its limitations. The study might be considered to be of small size and thus, caution should be exercised in drawing conclusions from the findings. All participants were anonymous and thus, any dropout analysis was impossible, but an approximate drop out analyses was performed suggesting a higher participation of elderly, those with a higher level of education and lower participation of women (2). Nonetheless, the study included all newly arrived refugees who participated in civic and health communication in the previously specified time period. In addition, it might be argued that the response rate is low, but in comparison with other studies in the field of migration and health (35) a response rate of 39.5% is to be considered rather good.

The primary limitations of the study are the cross-sectional nature of the data precluding statements of causality and also the single-items indicators for the measures of wellbeing. Also, PA were reported by the participants themselves and this may be a source of misclassification and bias the results. Self-reports are suitable to get information about PA among individuals, though there might be a risk that they either over-or underestimate their answers. It is also important to notice that the results presented in the current study should be seen in the light of that the majority of the participants were men (that might not be seen as a sample bias, but mirrors the composition of the newly arrived refugees), had a high educational level (more than 12 years) and were between 18 and 34 years. Also, the dependent variables sleep quality and stress only roughly record the underlying constructs and thus might be seen as potential limitations.

A strength of this study is at it was conducted closely after that the participants had received their permission to stay in Sweden, thus they had been in the country approximately between a few months and 2 years. The independent variables, PA, have earlier been used in Swedish public health surveys and are methodically tested concerning reliability and validity (36).

## Conclusion

Our findings demonstrate the importance of informing refugees of the relationship between wellbeing and PA and of how and where they can participate in PA. Multisectoral efforts are needed, both to increase refugees' awareness and to support their engagement in PA, regardless of their external circumstances. However, further research is needed to identify the specific barriers to and facilitators of PA among newly arrived refugees.

## Data Availability Statement

The datasets generated for this study are available on request to the corresponding author.

## Ethics Statement

This study was approved by the Regional Ethical Committee in Lund, Sweden (No 2014/2859). All participants received written information about the study and consented to participate by answering the questionnaire.

## Author Contributions

KSF, ML, and SZ contributed to the conception and design of the study. KSF was responsible for drafting the manuscript. MG and SZ were responsible for the analysis. EM, ML, MG, and SZ offered scientific suggestions. All authors contributed to the article and approved the submitted version.

## Conflict of Interest

The authors declare that the research was conducted in the absence of any commercial or financial relationships that could be construed as a potential conflict of interest.
